# Oral CBD-rich hemp extract modulates sterile inflammation in female and male rats

**DOI:** 10.3389/fphys.2023.1112906

**Published:** 2023-05-18

**Authors:** Shelby Hopkins, Tel Kelley, Rachel Roller, Robert S. Thompson, Dorothy B. Colagiovanni, Kris Chupka, Monika Fleshner

**Affiliations:** ^1^ Department of Integrative Physiology, University of Colorado at Boulder, Boulder, CO, United States; ^2^ Center for Neuroscience, University of Colorado at Boulder, Boulder, CO, United States; ^3^ Next Frontier Biosciences, Westminster, CO, United States

**Keywords:** cytokines, chemokines, corticosterone, glucose, stress proteins, edibles

## Abstract

**Introduction:** Cannabidiol (CBD) extract from the cannabis plant has biomedical and nutraceutical potential. Unlike tetrahydrocannabinol (THC), CBD products produce few psychoactive effects and pose little risk for abuse. There is emerging preclinical and clinical evidence that CBD is stress modulatory and may have anti-inflammatory properties. People across the United States legally ingest CBD-rich hemp extracts to manage mental and physical health problems, including stress and inflammation. Preclinical studies have revealed potential mechanisms for these effects; however, the impact of this prior work is diminished because many studies: 1) tested synthetic CBD rather than CBD-rich hemp extracts containing terpenes and/or other cannabinoids thought to enhance therapeutic benefits; 2) administered CBD via injection into the peritoneal cavity or the brain instead of oral ingestion; and 3) failed to examine potential sex differences. To address these gaps in the literature, the following study tested the hypothesis that the voluntary oral ingestion of CBD-rich hemp extract will attenuate the impact of stressor exposure on plasma and tissue inflammatory and stress proteins in females and males.

**Methods:** Adult male and female Sprague Dawley rats (10–15/group) were randomly assigned to be given cereal coated with either vehicle (coconut oil) or CBD-rich hemp extract (L-M0717, CBDrx/Functional Remedies, 20.0 mg/kg). After 7 days, rats were exposed to a well-established acute model of stress (100, 1.5 mA, 5-s, intermittent tail shocks, 90 min total duration) or remained in home cages as non-stressed controls.

**Results:** Stressor exposure induced a robust stress response, i.e., increased plasma corticosterone and blood glucose, and decreased spleen weight (a surrogate measure of sympathetic nervous system activation). Overall, stress-induced increases in inflammatory and stress proteins were lower in females than males, and oral CBD-rich hemp extract constrained these responses in adipose tissue (AT) and mesenteric lymph nodes (MLN). Consistent with previous reports, females had higher levels of stress-evoked corticosterone compared to males, which may have contributed to the constrained inflammatory response measured in females.

**Discussion:** Results from this study suggest that features of the acute stress response are impacted by oral ingestion of CBD-rich hemp extract in female and male rats, and the pattern of changes may be sex and tissue dependent.

## 1 Introduction

Cannabidiol (CBD) is a phytocannabinoid from the *Cannabis sativa* plant. Unlike tetrahydrocannabinol (THC), CBD is non-intoxicating, poses a low risk for abuse or dependency, and is generally well-tolerated (Bergamaschi et al., 2011; Iffland and Grotenhermen, 2017). Promising anecdotal and experimental evidence has increased consumer demand for CBD products. People across the United States legally ingest CBD-rich hemp extracts to manage anxiety, insomnia, and pain, all of which are exacerbated by stress and associated with inappropriate inflammation (Salim et al., 2012; Fleshner, 2013; Corroon and Phillips, 2018; Lurie, 2018; Devine et al., 2019; Wheeler et al., 2020; Moltke and Hindocha, 2021; Cavalcante et al., 2022; Uy et al., 2022). CBD-rich hemp products have complex and highly variable chemical compositions. For example, CBD_rx_/Functional Remedies (L-M0717) contains CBD and additional terpenes with putative bioactive and anti-inflammatory effects (Ciftci et al., 2011; Ciftci, Oztanir, and Cetin, 2014; Burcu et al., 2016). In addition, there is some evidence that CBD, terpenes, and other cannabinoids may synergize to produce greater *in vivo* physiological effects when taken together rather than separately (Gertsch, 2008; Rogerio et al., 2009; Russo, 2011; Zhong et al., 2014; Wang et al., 2019; Islam et al., 2020; Vieira et al., 2020; Jha et al., 2021; McDougall and McKenna, 2022). Finally, because consumers favor oral ingestion, many CBD-rich hemp products on the market are specifically formulated for oral consumption (Corroon and Phillips, 2018; Wheeler et al., 2020).

There is emerging evidence from *in vitro* and *in vivo* preclinical studies that CBD has biological, stress-modulatory, and anti-inflammatory properties (Malfait et al., 2000; Esposito et al., 2013; Li et al., 2013; Fogaça et al., 2014; Gallily et al., 2015; Elliott et al., 2018; Kim et al., 2022). While *in vitro* studies have revealed important mechanistic information, prior preclinical *in vivo* studies can be less compelling because most studies tested synthetic CBD rather than CBD-rich hemp extracts and administered CBD via injection into the peritoneal cavity or the brain, instead of oral ingestion (Schier et al., 2012; Nichols and Kaplan, 2020; Kim et al., 2022). These approaches do not reflect how people are using CBD products to manage the negative health consequences of stress and inappropriate inflammation such as anxiety, insomnia, and pain (Corroon and Phillips, 2018; Wheeler et al., 2020). In addition, more women than men report using oral CBD-rich hemp extracts to manage stress, making it important to include females and males in preclinical studies (Wheeler et al., 2020).

Inescapable tail shock (IS) is a model of acute stress that was chosen for this study because it robustly produces hallmark features of the biological stress response first described by Hans Selye, MD (Selye, 1985), including activation of the sympathetic nervous system (SNS), parasympathetic nervous system, and hypothalamic-pituitary-adrenal (HPA) axis (Fleshner et al., 1995b; Deak et al., 1999; Nguyen et al., 2000; Nickerson et al., 2006; Fleshner, 2013; Maslanik et al., 2013; Speaker et al., 2014). In addition to profound acute physiological responses, IS also produces negative system-wide consequences that persist for days to weeks after stressor cessation. For example, exposure to IS increases anxiety, disturbs sleep, diurnal rhythms, and the gut microbiota, and suppresses the generation of *in vivo* antibody responses to a benign protein (Maier, 1984; Fleshner et al., 1995a; Fleshner et al., 1996; Greenwood et al., 2005; Fleshner, 2011; Helmreich et al., 2012; Thompson et al., 2013; Greenwood et al., 2014; Speaker et al., 2014; Maier and Seligman, 2016; Thompson et al., 2016).

IS activates not only classical physiological stress responses but also stimulates peripheral sterile inflammation. Sterile inflammation is so named because signals driving this response are not foreign or pathogenic and include catecholamines and endogenous intracellular molecules that are released into the extracellular space, e.g., HMGB1, RNA, and Uric Acid Crystals (Johnson and Fleshner, 2006; Fleshner, 2013; Frank et al., 2013; Maslanik et al., 2013; Cox et al., 2014; Fleshner et al., 2017). It is feasible that stress-evoked sterile inflammation contributes to the exacerbation of mood and sleep disturbances as well as chronic pain (Kim and Jeon, 2018; Lurie, 2018; Won and Kim, 2020; Cavalcante et al., 2022; Palagini et al., 2022). If oral ingestion of CBD-rich hemp extract constrains stress-evoked inflammation, it may be a mechanism for the reported stress-modulatory effects of CBD.

Exposure to IS impacts many tissues and physiological systems. Heat shock protein 72 (Hsp72) is a ubiquitous intracellular stress protein that is upregulated by a broad range of cellular stressors and contributes to cell survival (Calderwood et al., 2007). Stressors reported to increase intracellular Hsp72 include heat, cold, shear force, hypoxia, and mitochondrial and oxidative stress (Gabai and Sherman, 2002; Campisi et al., 2003; Fleshner et al., 2004; Asea, 2005; Pespeni et al., 2005; Asea, 2007; Horowitz and Robinson, 2007; Henstridge et al., 2016). There is evidence that catecholamines, released by the SNS and the adrenal medulla, contribute to IS-induced increases in tissue cytokines and Hsp72 (Johnson et al., 2005). Elevated tissue concentrations of Hsp72, therefore, are indicative of general cellular and tissue distress.

Finally, few *in vivo* preclinical studies test the stress-modulatory impacts of oral CBD-rich hemp extract in both females and males. It is well established that there are sex differences in the neural, endocrine, and HPA-axis aspects of the stress response. For example, female rats compared to male rats exposed to IS had higher plasma corticosterone and lower Hsp72 in the pituitary gland, mesenteric lymph nodes (MLN), and liver but not the adrenal glands, spleen, and heart (Nickerson et al., 2006; Cox et al., 2014; Fonken et al., 2018). In addition, the pharmacokinetics and tissue distribution of oral CBD is sexually dimorphic. When compared to male rats, female rats had higher CBD concentrations in the liver and muscle but not in adipose tissue (Child and Tallon, 2022). Sex differences in these inflammatory modulatory pathways and CBD tissue distribution may contribute to sex and tissue specific effects of CBD.

The following study tested the hypothesis that the voluntary oral ingestion of CBD-rich hemp extract will attenuate the impact of stressor exposure on plasma and tissue inflammatory and stress proteins in females and males. No study to date has examined the effects of oral CBD-rich hemp extract on stress-evoked inflammatory proteins and Hsp72 measured in both the blood and stress responsive tissues in both female and male Sprague Dawley rats.

## 2 Methods and materials

### 2.1 Animals

All experimental protocols for this study were approved by the University of Colorado Animal Care and Use Committee, and special care was taken to ensure minimal animal discomfort during all procedures. Adult (PND 69–80 upon arrival) female and male Sprague Dawley rats (Envigo; Indianapolis, IN) were housed in a humidity and temperature (22°C) controlled environment and maintained on a 12:12 h light–dark cycle. Animals were individually housed in standard Nalgene Plexiglas cages (45.0 cm × 25.2 cm × 14.7 cm) with *ad libitum* access to food and water. Individual housing was necessary to ensure each animal was offered the correct dosing of CBD-rich hemp extract, which was corrected to each animal’s body weight.

### 2.2 Experimental design

**FIGURE 1:**
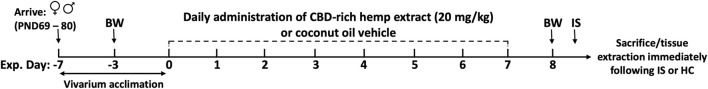
Experimental timeline. Female and male rats arrived at postnatal day (PND) 69–80, were singly housed, and allowed to acclimate to vivarium conditions for 7 days. Following acclimation, rats received daily administration of CBD-rich hemp extract in a coconut oil vehicle (20 mg/kg), or an equivalent volume of coconut oil vehicle, onto sugary cereal to eat *ad libitum*. Body weights (BW) were recorded on experimental days -3 and 8. Following 7 days of CBD-rich hemp extract or vehicle administration, rats were exposed to inescapable tail shock (IS) or remained within their home cage (HC) as non-stressed controls. All rats from all groups were sacrificed immediately following the IS procedure, and tissue and plasma were collected for later analysis.

The detailed experimental design is depicted in Figure 1. Upon arrival, rats were left undisturbed in their home cages for 1 week to acclimate to vivarium conditions. Female and male rats were then randomly assigned to receive daily doses of either CBD-rich hemp extract in a coconut oil vehicle or coconut oil vehicle without CBD-rich hemp extract. After 7 days, rats were randomly assigned to be exposed to inescapable tail shocks (Female Veh IS, *n* = 9; Female CBD IS, *n* = 9; Male Veh IS, *n* = 8; Male CBD IS, *n* = 12) or remained undisturbed in their home cages as unstressed controls (Female Veh HC, *n* = 8; Female CBD HC, *n* = 8; Male Veh HC, *n* = 7; Male CBD HC, *n* = 10). A 7-day administration protocol was chosen as there is some evidence supporting the accumulation of CBD in fatty tissues and in the plasma after dosing at multiple timepoints (Chiang and Rapaka, 1987; Devinsky et al., 2014; Taylor et al., 2018; Chayasirisobhon, 2020; Jewell et al., 2022). All animals from all groups were sacrificed immediately following the cessation of the IS procedure, and plasma and tissues were collected to analyze stress-related proteins.

### 2.3 CBD-rich hemp extract composition and administration

CBD-rich hemp extract in a coconut oil vehicle was supplied by CBD_Rx_/Functional Remedies (Boulder, CO). The composition of the extract is detailed in Table 1. The cannabinoid profile was characterized by Botanacor (Denver, CO) using high-performance liquid chromatography. Terpene profile was determined by ProVerde Laboratories (Milford, MA) using head-space gas chromatography. On a frosted mini wheat^®^ cereal, rats in the CBD-rich hemp extract group were offered 20.0 mg/kg cannabidiol suspended in a coconut oil vehicle while rats in the Veh group were offered an equivalent volume of coconut oil vehicle. Based on pilot testing, alternating cereal flavors increased cereal consumption. The appropriate volume of CBD-rich hemp extract or coconut oil vehicle was applied to the frosted side of an original- (days 1, 2, and 5) or chocolate- (days 3, 4, 6, and 7) flavored frosted mini wheats^®^ cereal and placed in the animal’s cage for *ad libitum* consumption. Because rats primarily eat and drink during their active cycle (Spiteri, 1982), the cereal was placed in the animal’s home cages before the onset of the active cycle. Experimenters visually examined all animal cages every day to monitor the daily consumption of cereal. These observations were recorded daily by the experimenters.

**TABLE 1:** Cannabinoid and terpene composition of the cannabidiol-rich hemp extract. The cannabinoid profile was determined using high-performance liquid chromatography; the terpene profile was determined using head-space gas chromatography.

Cannabinoid	Concentration
Cannabidiol (CBD)	46.8 mg/g
Tetrahydrocannabinol (THC)	2 mg/g
Cannabidiolic acid (CBDA)	0 mg/g
Tetrahydrocannabinolic acid (THCA)	0 mg/g
Cannabinol (CBN)	0 mg/g
Cannabigerol (CBG)	0 mg/g

Terpene	Concentration
B-caryophyllene	193 ppm
Myrcene	59 ppm
Humulene	37 ppm
A-bisabolol	34 ppm
Guaiol	25 ppm
Limonene	23 ppm
Terpineol	11 ppm
Caryophyllene oxide	10 ppm
Borneol	6 ppm
Isopulegol	5 ppm
Nerolidol-trans	5 ppm
Linalool	4 ppm

### 2.4 Inescapable tail shock (IS) protocol

Rats in the IS group were restrained in Plexiglas tubes (23.4 cm × 7 cm) with their tails protruding out of the tube. Electrodes were placed over the tail and 100, 5-s, 1.5 mA shocks were administered intermittently by an automated shock system (Precision Calculated Animal Shocker, Colbourn Instruments) over approximately 90 min. Immediately following cessation of the stressor protocol, all rats from all groups were euthanized, and blood and tissues were collected. This is an optimal time point for capturing the immediate effects of the acute stress response and the exaggerated sterile inflammatory response that is evoked by this IS protocol (Campisi and Fleshner, 2003; Maslanik et al., 2012).

### 2.5 Sacrifice and tissue collection

Immediately following stressor termination, all animals from all groups were rapidly decapitated and trunk blood was collected into EDTA tubes. Blood was then centrifuged at 3,000 rpm for 15 min and plasma was collected and divided into aliquots. Blood glucose measurements were taken from trunk blood using a glucometer and glucose test strips (Contour Next). Spleen, liver (left medial lobe nearest to the portal vasculature), thymus, MLNs, and subcutaneous adipose tissue (AT), were aseptically dissected, weighed (spleen and thymus), collected into polypropylene tubes, and snap frozen in liquid nitrogen. All samples were stored at −80°C.

### 2.6 Plasma CBD and COOH-CBD

Plasma concentration of CBD and COOH-CBD, a metabolite of CBD, were measured by Next Frontier Biosciences (Westminster CO) using LC-QTOF mass spectrometry. Relative concentrations were calculated based on the intensity ratio of the COOH-CBD to an internal standard (CBD-d3).

### 2.7 Plasma corticosterone and inflammatory protein assays

Plasma corticosterone was measured using a commercial corticosterone enzyme-linked immunosorbent assay kit (ELISA; Arbor Assays, Ann Arbor, MI, intra-assay precision 11%, inter-assay precision 15.1%). Heat extraction was used to degrade corticosterone-binding protein. Plasma was diluted 1:50. Plasma Cytokine-Induced Neutrophil Chemoattractant-1 (CINC-1, intra-assay precision 5.6%, inter-assay precision 6.3%), Interleukin (IL-)1beta (IL-1β, intra-assay precision 3.9%, inter-assay precision 4.4%), IL-6 (intra-assay precision 4.5%, inter-assay precision 7%), and IL-10 (intra-assay precision 3%, inter-assay precision 7.1%) were measured using individual commercial sandwich ELISAs (R&D systems, Minneapolis, MN). Plasma was diluted 1:25 for CINC-1 and run neat for IL-1β, IL-6, and IL-10. All ELISAs were utilized in accordance with the manufacturer’s instructions. Optical densities were measured using a SpectraMax Plus 354 plate reader (Molecular Devices, Sunnyvale, CA) at wavelengths specified in product manuals and analyzed using SOFTMAX PRO software.

### 2.8 Tissue homogenization

MLNs, AT, spleen, and liver samples were weighed using a digital scale (Sartorius R200D) and the volume of radioimmunoprecipitation (RIPA) lysis buffer was adjusted to a ratio of 3.0 mL of buffer per gram of tissue to correct for total protein concentrations. Tissue samples were then added to ice-cold RIPA lysis buffer (0.5 M Tris–HCl, pH 7.4, 1.5 M NaCl, 2.5% deoxycholic acid, 10% NP-40, 10 mM EDTA, 1 mM NaF, 1.0 mM sodium orthovanadate, 1.0 mM phenylmethylsulfonylfluoride) containing protease and phosphatase inhibitors and 0.01% phosphatase inhibitor cocktail (protease inhibitor cocktail tablet, Roche, Indianapolis, IN; 0.01% phosphatase inhibitor cocktail, Sigma, St. Louis, MO). Tissue samples were homogenized with ceramic beads (2 s × 50 s at 5,000 rpm) using a Precellys 24 high-throughput tissue homogenizer (Bertin Corp, Rockville, MD); and lysates were divided into aliquots and stored at −80°C for later analysis.

### 2.9 Tissue Hsp72 and inflammatory proteins assays

High-sensitivity ELISA kits were used according to the manufacturer’s instructions to measure tissue Hsp72 (Enzo Life Sciences, intra-assay precision <5%, inter-assay precision <13%), CINC-1, IL-1β, IL-6, and IL-10 (R&D Systems, Minneapolis, MN). Optical densities were measured using a SpectraMax Plus 354 plate reader. MLN lysates were diluted 1:4 for Hsp72, neat for CINC-1, and 1:2 for IL-1β, IL-6, and IL-10. AT lysates were diluted 1:4 for Hsp72 and 1:2 for CINC-1, IL-1β, IL-6, and IL-10. Spleen lysates were diluted 1:4 for Hsp72; 1:2 for CINC-1; 1:8 for IL-1β; 1:2 for IL-6; and 1:4 for IL-10. Liver lysates were diluted 1:4 for Hsp72; 1:25 for the CINC-1 stress group, and CINC-1 controls were run neat.

### 2.10 Tissue homogenate protein assays

Total protein from the liver, spleen, MLNs, and AT homogenates was measured via bicinchoninic acid assay (Thermo Scientific, Waltham, MA) according to the manufacturer’s instructions. For protein quantification, homogenates were diluted at 1:10 for the MLNs and AT, 1:40 for the spleen, and 1:100 for the liver. Total protein was used to normalize levels of Hsp72 and inflammatory proteins of each of the tissue homogenates for comparison.

### 2.11 Statistical analyses

A non-parametric Kruskal-Wallis test, followed by Dunn’s *post hoc* analysis, was used to compare total cereal consumption between groups. A Mann-Whitney non-parametric *post hoc* test was then used to compare CBD-coated cereal consumption between females and males. A two-factor ANOVA was used to determine the impact of CBD-rich hemp extract (Veh vs. CBD) on body weight gain in female and male rats. An unpaired t-test (female vs. male) was used to determine the impact of CBD-rich hemp extract consumption on the relative concentration of CBD and COOH-CBD in the plasma in females and males. A three-factor ANOVA was used to evaluate the effect of voluntary ingestion of CBD-rich hemp extract (Veh vs. CBD) on the acute stress response (HC vs. IS) between females and males (female vs. male). *Post-hoc* pairwise comparisons using Fisher’s least significant difference test were performed when two or more main effects or any interaction effect was observed. Sample data are presented as means ± the standard error of the mean. In all cases, an alpha set at *p* < 0.05 was considered statistically significant. GraphPad Prism version 9.5.1 for Apple, GraphPad Software, San Diego, California United States, www.graphpad.comwas used for statistical tests and data presentation.

## 3 Results

### 3.1 Cereal consumption and body weight

#### 3.1.1 Cereal consumption

**FIGURE 2:**
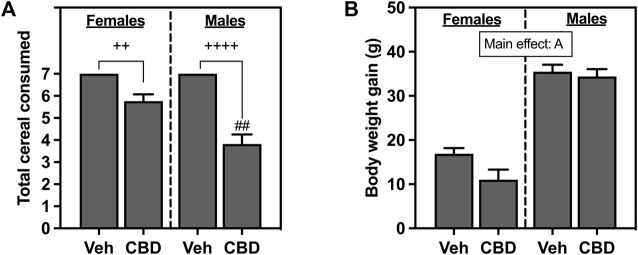
**(A)** Total voluntary consumption of coconut oil vehicle (Veh) or CBD-rich hemp extract (CBD) treated cereal pieces and **(B)** body weight gain over the 7-day administration period. All female and male rats assigned to the vehicle group consumed every piece of coconut oil-treated cereal they were offered. Females are graphed to the left of the dashed line; males are graphed to the right of the dashed line. Data are presented as mean ± SEM; A*p* < 0.05 (main effect of sex); ^##^ *p* < 0.01 (Mann-Whitney post hoc effect of Sex); ^++^ *p* < 0.01, ^++++^ *p* < 0.0001 (Dunn’s *post hoc* effect of CBD).

Total cereal consumption for female and male rats is shown in Figure 2A. All rats in the Veh groups ate every piece of cereal that was administered daily for 7 days. Based on the Kruskal-Wallis non-parametric comparisons and Dunn’s *post hoc* comparison, both female (*p* = 0.0095) and male rats (*p* < 0.0001) offered cereal coated with CBD-rich hemp extract consumed fewer pieces of cereal than rats offered Veh coated cereal. Based on the non-parametric Mann-Whitney *post hoc* test, males as compared to females consumed fewer pieces of CBD-rich hemp extract coated cereal (*p* = 0.0024). The emergence of this unexpected sex difference in the ingestion of CBD-rich hemp extract coated cereal could confound our other outcomes. To control for this, we performed secondary matching analyses for any outcome that had statistically reliable interaction effects that included sex (CBD x sex or CBD x sex x IS) revealed by multifactorial ANOVA (Thomas et al., 2020). Females and males from the CBD-rich hemp extract groups were matched for cereal consumption. These female and male CBD groups were used in all secondary analyses of matched CBD-rich hemp cereal consumption groups, when appropriate. Based on Kruskal-Wallis non-parametric comparisons and non-parametric *post hoc* tests, matching analyses eliminated the sex difference in CBD-rich hemp cereal consumption (Supplementary Figure S1A, *n* = 5 per group).

#### 3.1.2 Body weight

Body weight gain for female and male rats that ingested CBD-rich hemp extract or Veh-treated cereal is shown in Figure 2B. Female and male rats gained body weight throughout the experiment, with analysis revealing that male rats gained significantly more weight than female rats (*F*_1, 65_ = 138.2, *p* < 0.0001).

### 3.2 Plasma CBD and COOH-CBD

**FIGURE 3:**
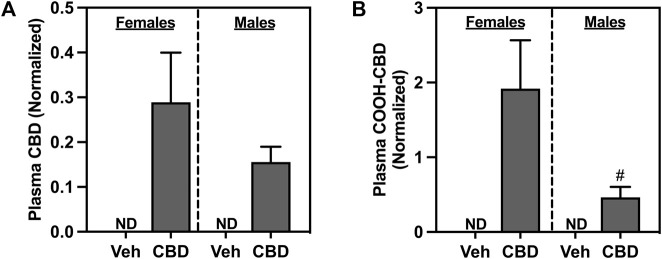
Concentrations of normalized plasma **(A)** cannabidiol (CBD), and **(B)** COOH-CBD, a metabolite of CBD. Females are graphed to the left of the dashed line; males are graphed to the right of the dashed line. Data are presented as mean ± SEM; Symbols: ^#^ *p* < 0.05 (unpaired T-test effect, female vs male); ND = not detectable.

Oral ingestion of CBD-rich hemp extracts increased plasma concentration of CBD (Figure 3A) and COOH-CBD (Figure 3B) in both females and males. Females had higher plasma levels of COOH-CBD than males (*t*_37_ = 2.476, *p* = 0.0180). Neither CBD nor COOH-CBD was detectable (ND) in the plasma of Veh rats. Analysis of the matched CBD-rich hemp cereal consumption groups eliminated sex differences in plasma concentrations of CBD or COOH-CBD (Supplementary Figures S1B, C).

### 3.3 Effect of voluntary CBD-rich hemp extract ingestion on the acute stress response

**FIGURE 4:**
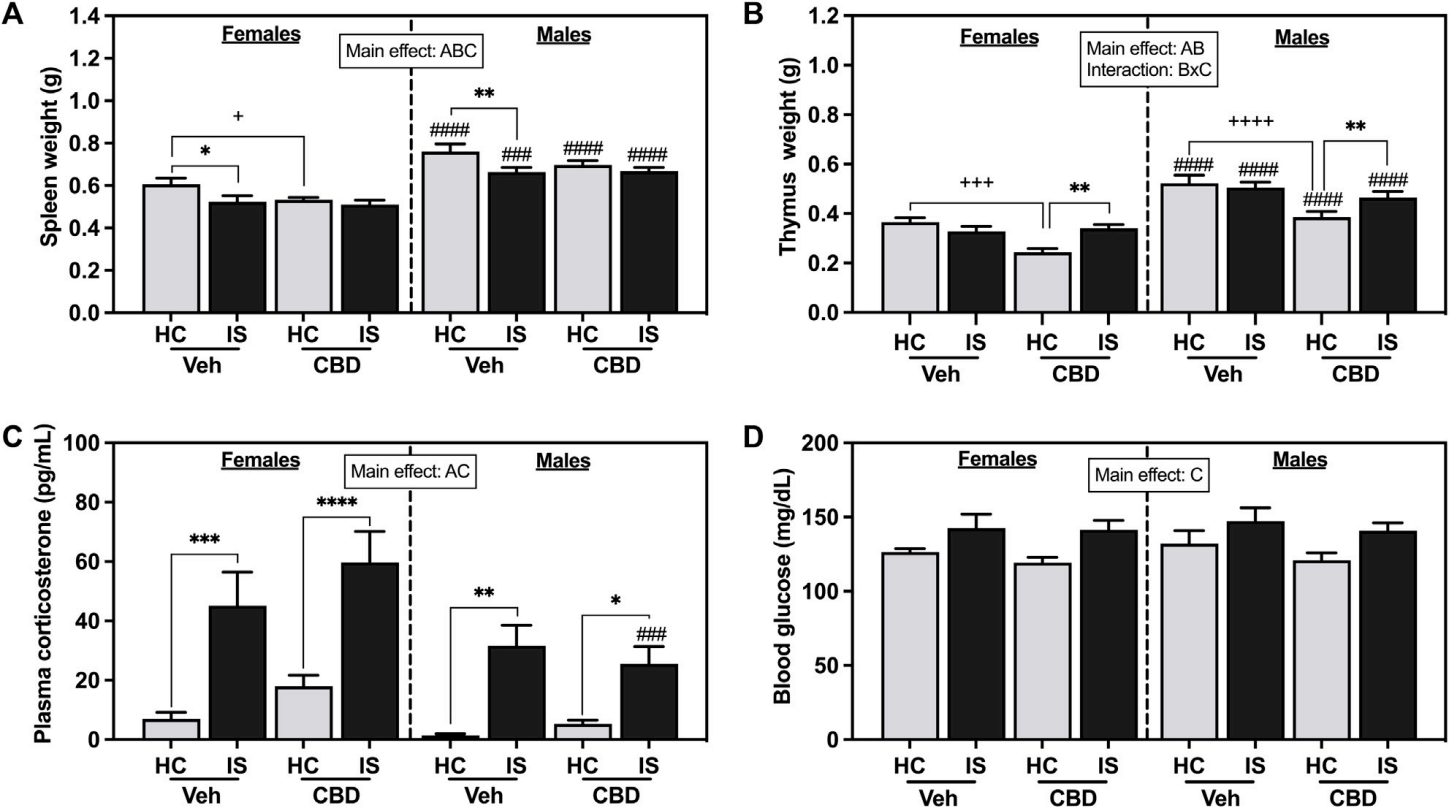
**(A)** Spleen weight, **(B)** thymus weight, **(C)** plasma corticosterone, and **(D)** blood glucose. Females are graphed to the left of the dashed line; males are graphed to the right of the dashed line. Data are presented as mean ± SEM; Symbols: ^A^ *p* < 0.05 (main effect of sex); ^B^ *p* < 0.05 (main effect of CBD); ^C^ *p* < 0.05 (main effect of IS); ^###^ *p* < 0.001, ^####^ *p* < 0.0001 (Fisher’s LSD *post hoc* effect of Sex); ^+^ *p* < 0.05, ^++++^ *p* < 0.0001, ^++++^ *p* < 0.0001 (Fisher’s LSD *post hoc* effect of CBD); *p < 0.05, **p < 0.01, ***p < 0.001, ****p < 0.0001 (Fisher’s LSD post hoc effect of IS). Vehicle group = Veh, Cannabidiol-rich hemp extract group = CBD, unstressed home cage controls = HC, inescapable tail shock = IS.

The effects of CBD-rich hemp extract and IS exposure on spleen weight are illustrated in Figure 4A. Male rats had heavier spleens than females (*F*_1, 63_ = 84.94, *p* < 0.0001). Oral ingestion of CBD-rich hemp reduced spleen weight (*F*_1, 63_ = 4.777, *p* = 0.0326). IS decreased spleen weight in females and males (*F*_1, 63_ = 11.79, *p* = 0.0011). *Post hoc* analysis revealed CBD-rich hemp extract decreased spleen weight in the female HC group as compared to Veh (*p* = 0.0401); and IS decreased spleen weight in the Veh group in both females (*p* = 0.0180) and males (*p* = 0.0097) as compared to HC groups. Effects found in thymus weight are depicted in Figure 3B. Thymus mass was greater in males than females (*F*_1, 62_ = 88.03, *p* < 0.0001). A main effect of CBD-rich hemp extract on thymus weight was observed (*F*_1, 62_ = 20.25, *p* < 0.0001). In addition, an interaction between CBD-rich hemp extract and IS on thymus weight (*F*_1, 62_ = 12.68, *p* = 0.0007) was observed. *Post hoc* analysis revealed that female and male rats in the CBD-rich hemp extract groups had lower thymus weight than Veh groups (females, *p* = 0.0007; males, *p* < 0.0001); and IS increased thymus weight in both female and male CBD groups (females, *p* = 0.0038; males, *p* = 0.0070).

The effects of IS on plasma corticosterone are shown in Figure 3C. IS exposure increased levels of circulating corticosterone in both females and males (*F*_1, 63_ = 45.64, *p* < 0.0001). Stressor exposure evoked a greater response in females compared to males (*F*_1, 63_ = 11.67, *p* = 0.0011). IS significantly increased blood glucose (Figure 3D, *F*_1, 63_ = 15.35, *p* = 0.0002). CBD-rich hemp extract ingestion did not affect stress-associated increases in plasma corticosterone or blood glucose.

### 3.4 Effect of CBD-rich hemp extract ingestion on measures of stress and sterile inflammation

**FIGURE 5:**
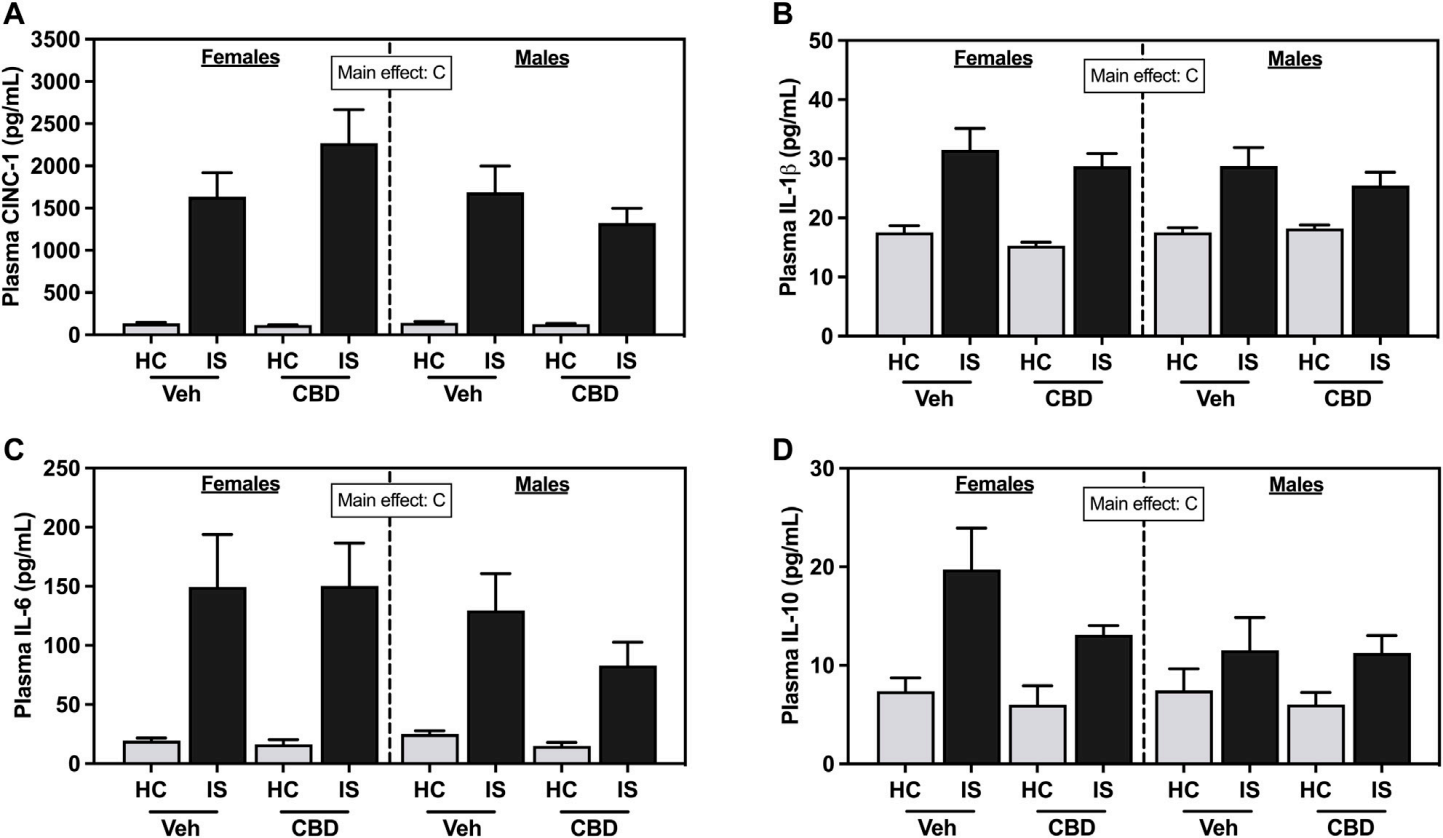
Plasma concentrations of **(A)** Cytokine-Induced Neutrophil Chemoattractant 1 (CINC-1), **(B)** Interleukin- (IL-) 1β, **(C)** IL-6, and **(D)** IL-10. Females are graphed to the left of the dashed line; males are graphed to the right of the dashed line. Data are presented as mean ± SEM; Symbols: ^C^ *p* < 0.05 (main effect of IS). Vehicle group = Veh, Cannabidiol-rich hemp extract group = CBD, unstressed home cage controls = HC, inescapable tail shock = IS.

IS increased plasma CINC-1 (Figure 5A, *F*_1, 63_ = 104.7, *p* < 0.0001), IL-1β (Figure 5B, *F*_1, 63_ = 54.12, *p* < 0.0001), IL-6 (Figure 5C, *F*_1, 63_ = 37.68, *p* < 0.0001), and IL-10 (Figure 5D, *F*_1, 63_ = 18.62, *p* < 0.0001) in female and male rats. CBD-rich hemp extract had no effect.

**FIGURE 6:**
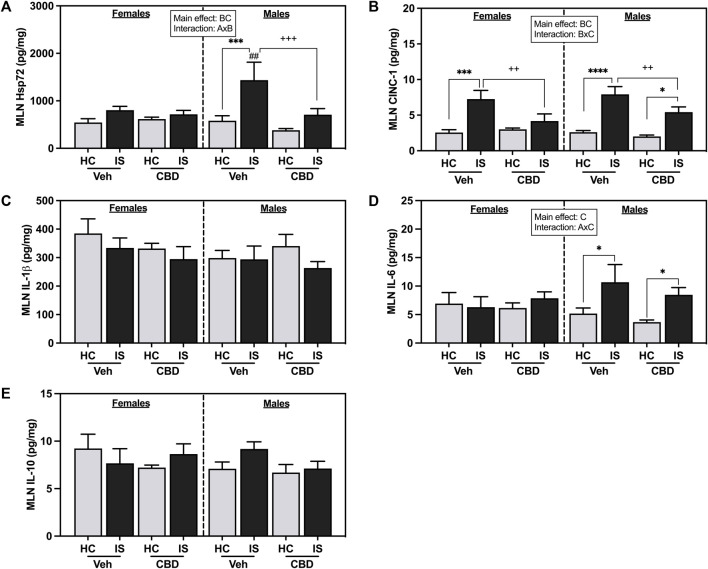
Mesenteric lymph node (MLN) concentrations of **(A)** CINC-1, **(B)** Heat shock protein 72 (Hsp72), **(C)** IL-1β, **(D)** IL-6, and **(E)** IL-10. Females are graphed to the left of the dashed line; males are graphed to the right of the dashed line. Data are presented as mean ± SEM; Symbols: ^A^ *p* < 0.05 (main effect of sex); ^B^ *p* < 0.05 (main effect of CBD); ^C^ *p* < 0.05 (main effect of IS); ^##^ *p* < 0.01(Fisher’s LSD *post hoc* effect of Sex); ^++^ *p* < 0.01, ^+++^ *p* < 0.001 (Fisher’s LSD *post hoc* effect of CBD); *p < 0.05, ***p < 0.001, ****p < 0.0001 (Fisher’s LSD post hoc effect of IS). Vehicle group = Veh, Cannabidiol-rich hemp extract group = CBD, unstressed home cage controls = HC, inescapable tail shock = IS.

IS reliably increased CINC-1 in the MLNs of females and males (IS (*F*_1, 53_ = 41.36, *p* < 0.0001) and CBD-rich hemp extract reduced that effect (Figure 6A). There was a reliable interaction between CBD-rich hemp extract and IS for CINC-1 concentration in the MLNs (*F*_1, 53_ = 5.766, *p* = 0.0199), with *post hoc* analysis revealing that IS increased CINC-1 in the MLNs of female (*p* = 0.0003) and male (*p* < 0.0001) rats; and the voluntary ingestion of CBD-rich hemp extract attenuated the stress-induced increase in CINC-1 in the MLNs of both female (*p* = 0.0082) and male (*p* = 0.0183) rats.

IS increased overall Hsp72 in the MLNs (*F*_1, 58_ = 13.85, *p* = 0.0004) and CBD-rich hemp extract decreased overall Hsp72 levels (*F*_1, 58_ = 5.160, *p* = 0.0268). An interaction between sex and CBD-rich hemp extract was found in Hsp72 in the MLNs (*F*_1, 58_ = 4.835, *p* = 0.0319). *Post hoc* analysis revealed that IS exposure increased Hsp72 in the MLNs of male Veh rats only (*p* = 0.0005, Figure 6B). The stress-induced increase in Hsp72 in the MLNs of males was attenuated by the voluntary ingestion of CBD-rich hemp extract (*p* = 0.0005). Secondary analysis of matched CBD-rich hemp cereal consumption groups revealed main effects of IS (*F*_1, 40_ = 6.841, *p* = 0.0125) and CBD (*F*_1,40_ = 5.188, *p* = 0.0282) on Hsp72 in the MLNs; and consistent with previous analyses, ingestion of CBD-rich hemp extract attenuated the IS-induced increase in MLN Hsp72 in males (*p* = 0.0028, Supplementary Figure S1D).

A main effect of IS (*F*_1, 52_ = 6.684, *p* = 0.0126) and an interaction between sex and IS was found for IL-6 concentration in the MLNs (*F*_1, 52_ = 4.389, *p* = 0.0411). *Post hoc* analysis revealed that IS increased MLN IL-6 in both Veh and CBD groups in males but not females (Veh, *p* = 0.0271; Figure 6D). No effects were found in the MLNs for IL-1β (Figure 6C) and L-10 (Figure 6E).

**FIGURE 7:**
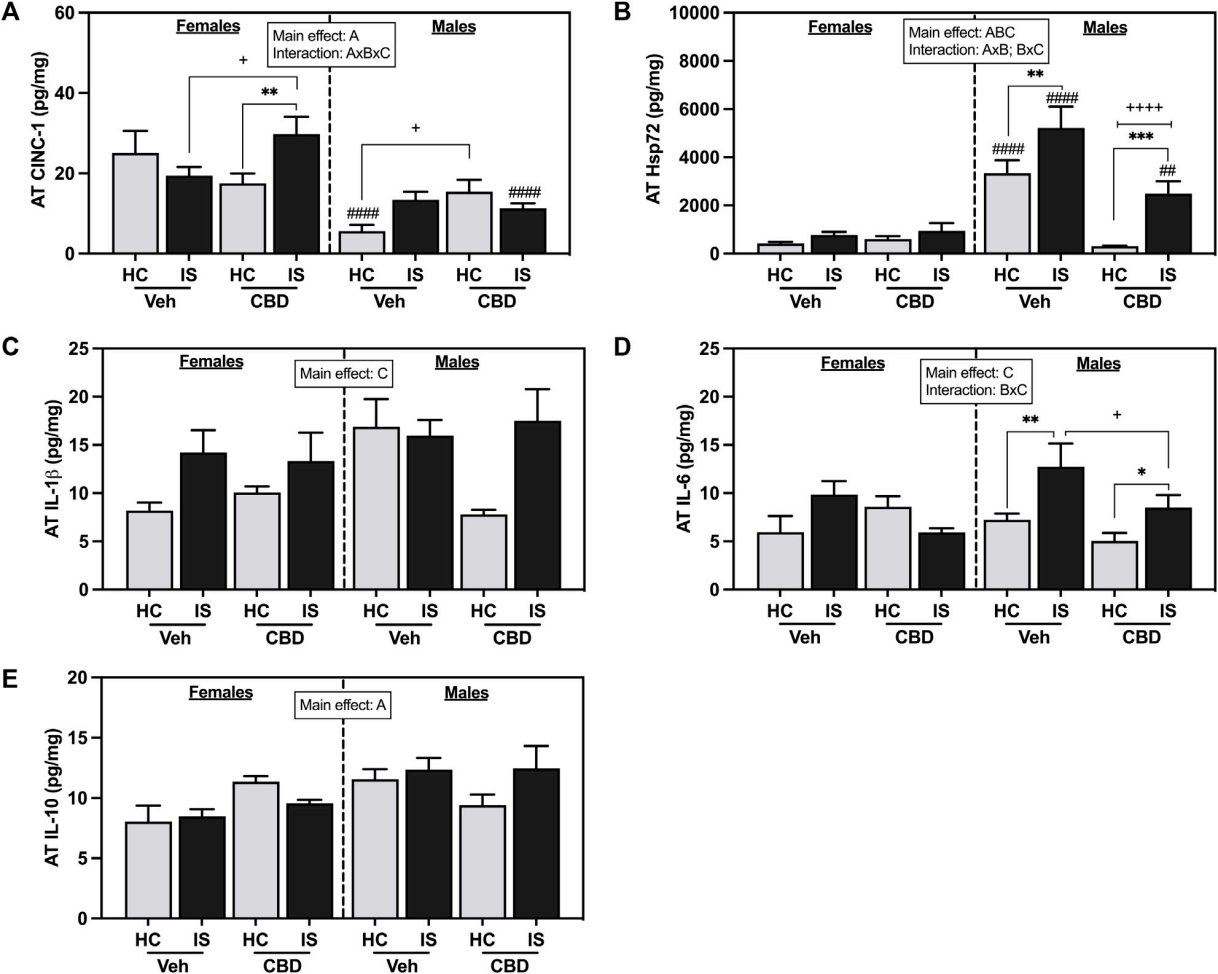
Adipose tissue concentrations of **(A)** CINC-1, **(B)** Hsp72, **(C)** IL-1β, **(D)** IL-6, and **(E)** IL-10. Females are graphed to the left of the dashed line; males are graphed to the right of the dashed line. Data are presented as mean ± SEM; Symbols: ^A^ *p* < 0.05 (main effect of sex); ^B^ *p* < 0.05 (main effect of CBD); ^C^ *p* < 0.05 (main effect of IS); ^##^ *p* < 0.01, ^####^ *p* < 0.0001 (Fisher’s LSD post hoc effect of Sex); ^+^ *p* < 0.05, ^++++^ *p* < 0.0001 (Fisher’s LSD post hoc effect of CBD); *p < 0.05, **p < 0.01, ***p < 0.001 (Fisher’s LSD *post hoc* effect of IS). Vehicle group = Veh, Cannabidiol-rich hemp extract group = CBD, unstressed home cage controls = HC, inescapable tail shock = IS.

Female rats had higher concentrations of CINC-1 in AT (Figure 7A, *F*_1, 62_ = 28.45, *p* < 0.0001). A three-way interaction between sex, CBD, and IS was found (*F*_1, 62_ = 11.88, *p* = 0.0010), with *post hoc* analyses showing that AT CINC-1 was reliably increased in the female CBD-rich hemp extract IS group compared to CBD-rich hemp extract HC group (*p* = 0.0083) and Veh IS groups (*p* = 0.0166). The male rats that consumed CBD-rich hemp extract had significantly higher concentrations of CINC-1 than the male Veh group (*p* = 0.0295). Male Veh HC (*p* < 0.0001) and CBD-rich hemp extract IS (*p* = 0.0004) rats had significantly lower levels of CINC-1 in AT as compared to females. The results from our secondary analysis of matched CBD-rich hemp cereal consumption groups mirrored the primary ANOVA findings. There was a reliable main effect of sex (*F*_1, 43_ = 24.59, *p* < 0.0001) and a three-way interaction effect between sex, CBD, and stress (*F*_1, 43_ = 8.909, *p =* 0.0047, Supplementary Figure S1E). *Post hoc* analyses were unchanged in females, revealing an effect of IS only in the females that consumed CBD-rich hemp extract (*p* = 0.0195). The females exposed to IS that also consumed CBD had significantly higher AT CINC-1 than the female Veh IS group (*p* = 0.0159). The effect of CBD-rich hemp extract seen in the HC groups of the male rats in the initial analyses, however, was no longer reliable.

Male rats had higher levels of Hsp72 in the AT as compared to females (Figure 7B, *F*_1, 62_ = 48.92, *p* < 0.0001). CBD-rich hemp extract consumption decreased overall levels of Hsp72 in AT (*F*_1, 62_ = 19.30, *p* < 0.0001) while IS increased Hsp72 (*F*_1, 62_ = 14.90, *p* = 0.0003). Interaction effects of sex and CBD-rich hemp extract (*F*_1, 62_ = 24.59, *p* < 0.0001) and sex and IS (*F*_1, 62_ = 7.431, *p* = 0.0083) were found with *post hoc* analysis showing that only males had an increase in Hsp72 in response to IS (Veh, *p* = 0.0057; CBD-rich hemp extract, *p* = 0.0002), and this effect was reduced by the consumption of CBD-rich hemp extract (*p* < 0.0001). Results of the secondary analysis of matched CBD-rich hemp cereal consumption groups were consistent with the primary findings. We found reliable main effects of sex (*F*_1, 43_ = 31.97, *p* < 0.0001), CBD (*F*_1, 43_ = 18.78, *p* < 0.0001), and stress (*F*_1, 43_ = 7.799, *p* = 0.0078); and an interaction between sex and CBD (*F*_1, 43_ = 17.87, *p* = 0.0001, Supplementary Figure S1F). *Post hoc* analysis revealed that IS increased Hsp72 in males but not females, and CBD constrained that effect.

IS increased AT IL-1β (Figure 7C, *F*_1, 59_ = 6.637, *p* = 0.0125) and IL-6 (Figure 7D, *F*_1, 42_ = 5.797, *p* = 0.0205). An interaction between CBD-rich hemp extract and IS was seen for AT IL-6 (*F*_1, 42_ = 4.133, *p* = 0.0484). *Post hoc* analysis revealed that voluntary ingestion of CBD-rich hemp extract reduced the stress-induced increase in IL-6 seen in the AT of male rats (*p* = 0.0233); and IS exposure increased IL-6 in the AT of male rats only (Veh, *p* = 0.0071; CBD-rich hemp extract, *p* = 0.0433). A main effect of sex was seen in the concentration of IL-10 in the AT, with male rats having higher overall values (Figure 7E, *F*_1, 42_ = 4.914, *p* = 0.0321).

**FIGURE 8:**
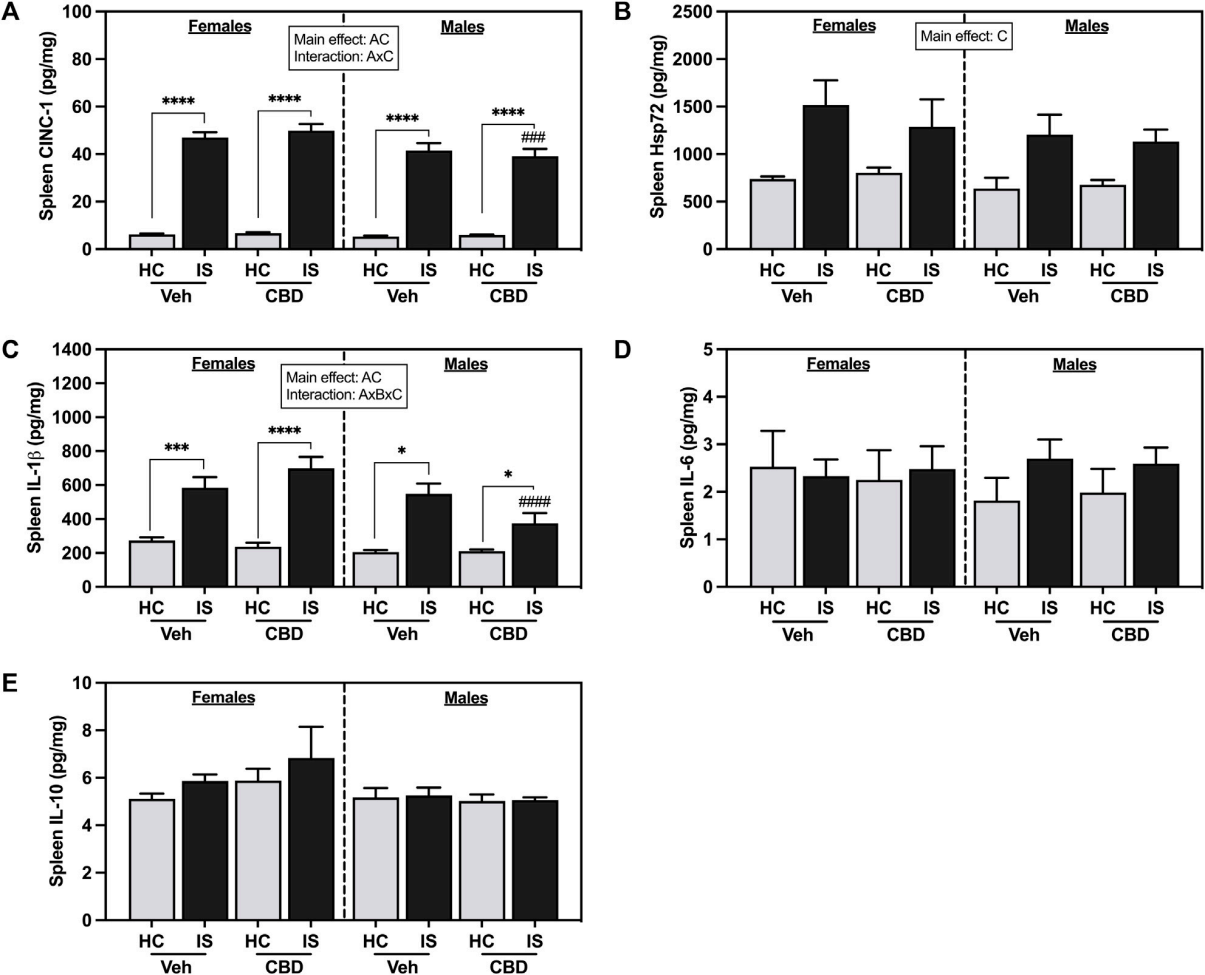
Spleen concentrations of **(A)** CINC-1, **(B)** Hsp72, **(C)** IL-1β, **(D)** IL-6, and **(E)** IL-10. Females are graphed to the left of the dashed line; males are graphed to the right of the dashed line. Data are presented as mean ± SEM; Symbols: ^A^ *p* < 0.05 (main effect of sex); ^B^ *p* < 0.05 (main effect of CBD); ^C^ *p* < 0.05 (main effect of IS); ^###^ *p* < 0.001, ^####^ *p* < 0.0001 (Fisher’s LSD *post hoc* effect of Sex); *p < 0.05, ***p < 0.001, ****p < 0.0001 (Fisher’s LSD *post hoc* effect of IS). Vehicle group = Veh, Cannabidiol-rich hemp extract group = CBD, unstressed home cage controls = HC, inescapable tail shock = IS.

IS increased splenic CINC-1 (Figure 8A, *F*_1, 62_ = 601.1, *p* < 0.0001) and Hsp72 (Figure 8B, *F*_1, 62_ = 22.93, *p* < 0.0001). A main effect of sex revealed that males had overall lower levels of CINC-1 (*F*_1, 62_ = 8.237, *p* = 0.0056). There was a reliable interaction between sex and IS (*F*_1, 62_ = 5.364, *p* = 0.0239). *Post hoc* analysis showed that males that consumed CBD-rich hemp extract and were exposed to IS had decreased CINC-1 as compared to females (*p* = 0.0005).

Males had overall lower spleen IL-1β levels than females (Figure 8C, *F*_1, 57_ = 9.718, *p* = 0.0029). There was a main effect of IS, and a three-way interaction between sex, CBD-rich hemp extract, and IS (*F*_1, 57_ = 5.154, *p* = 0.0270). *Post hoc* analysis showed that both females (Veh, *p* = 0.0001; CBD-rich hemp extract, *p* < 0.0001) and males (Veh, *p* < 0.0001; CBD-rich hemp extract, *p* = 0.0125) had increased IL-1β due to IS, with stressed males that consumed CBD-rich hemp extract having significantly lower levels than females (*p* < 0.0001). Consistent with the primary analyses, secondary analyses of the matched CBD-rich hemp cereal consumption groups revealed main effects of sex (*F*_1, 40_ = 7.745, *p* = 0.0082) and stress (*F*_1, 40_ = 65.61, *p* < 0.0001, Supplementary Figure S1G). The three-way interaction between sex, CBD-rich hemp extract, and IS was no longer reliable. *Post hoc* analyses mirrored the results from the primary analysis, showing that IS increased splenic IL-1β in females (Veh, *p* < 0.0001; CBD-rich hemp extract, *p* < 0.0001) and males (Veh, *p* < 0.0001; CBD-rich hemp extract, *p* = 0.0213). No effects were found in the spleen for IL-6 (Figure 8D) or IL-10 (Figure 8E).

**FIGURE 9:**
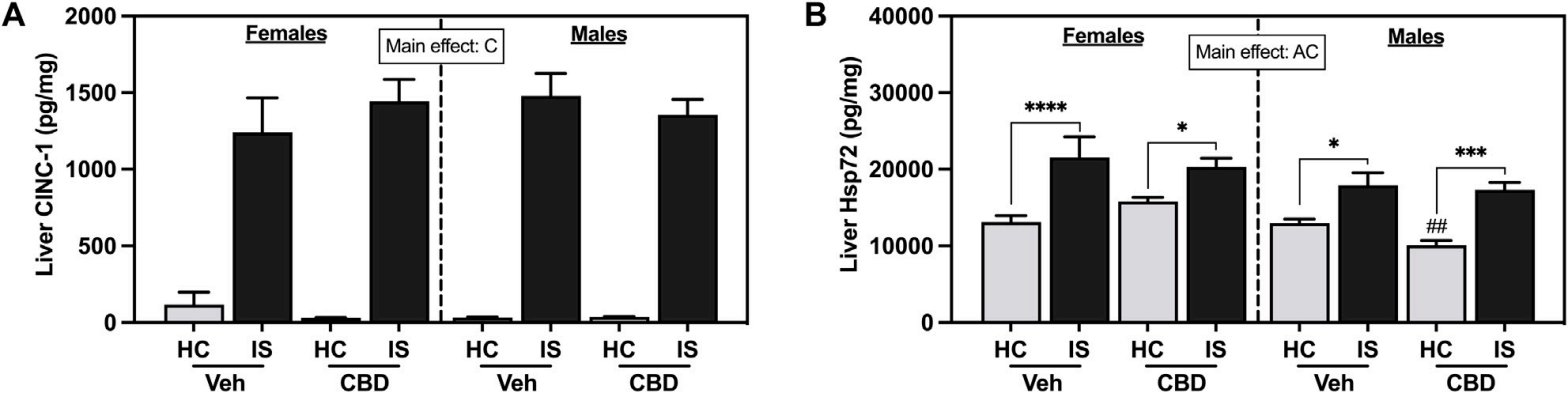
Liver concentrations of **(A)** CINC-1 and **(B)** Hsp72. Females are graphed to the left of the dashed line; males are graphed to the right of the dashed line. Data are presented as mean ± SEM; Symbols: ^A^ *p* < 0.05 (main effect of sex); ^C^ *p* < 0.05 (main effect of IS); ^##^ *p* < 0.01 (Fisher’s LSD post hoc effect of sex); *p < 0.05, ***p < 0.001, ****p < 0.0001 (Fisher’s LSD post hoc effect of IS). Vehicle group = Veh, Cannabidiol-rich hemp extract group = CBD, unstressed home cage controls = HC, inescapable tail shock = IS.

IS increased liver CINC-1 (Figure 9A, *F*_1, 59_ = 232.2, *p* < 0.0001) and Hsp72 (Figure 9B, *F*_1, 60_ = 40.87, *p* < 0.0001). There was also a main effect of sex for liver Hsp72 (*F*_1, 60_ = 10.12, *p* = 0.0023). *Post hoc* analysis confirmed that IS increased liver Hsp72 in both females (Veh, *p* < 0.0001; CBD-rich hemp extract, *p* = 0.0287) and males (Veh, *p* = 0.0199; CBD-rich hemp extract, *p* = 0.0001) and males that consumed CBD-rich hemp extract in the Veh group had lower Hsp72 in the liver as compared to females (*p* = 0.0044).

## 4 Discussion

Recent surveys of older and younger adults concluded that most CBD users believe there is compelling experimental evidence that CBD-rich hemp products are both safe and effective even though results from rigorously designed studies are equivocal (Astray et al., 2022; Garcia-Romeu et al., 2022; Kaufmann et al., 2022; Kimless et al., 2022; Utter et al., 2022; Wysota et al., 2022). There remains a need, therefore, for additional clinical trials and preclinical studies that more closely reflect the route of administration and phytochemical composition of CBD products being used. Preclinical studies that explore the stress-modulatory impacts of CBD are necessary to determine the distribution and potential cumulative impacts of ingesting CBD-rich hemp extracts in tissues. The current study tested if daily oral ingestion of CBD-rich hemp extract impacts inflammatory and cellular stress proteins in plasma and tissues. To the best of our knowledge, no study to date has focused on the effect of oral CBD-rich hemp extract on the *in vivo* sterile inflammatory response evoked by an intense, acute stressor in female and male rats. Overall, oral CBD-rich hemp extract and acute traumatic stress impact inflammatory proteins and Hsp72 in a tissue specific and sex-dependent way.

The inescapable tail shock (IS) protocol utilized in this experiment is a well-studied model of acute traumatic stress that evokes sterile inflammation without inflicting tissue damage (Fleshner et al., 1995a; Brennan et al., 1996; Deak et al., 1997; Nguyen et al., 1998; Deak et al., 1999; Nguyen et al., 2000; Moraska et al., 2002; Campisi and Fleshner, 2003; Greenwood et al., 2003; Greenwood et al., 2005; Campisi et al., 2012; Fleshner, 2013; Frank et al., 2013; Beninson et al., 2014; Frank et al., 2016; Fleshner et al., 2017). Consistent with results from these previous studies, IS activated the HPA-axis and decreased spleen weight, which is indicative of SNS activation (Domínguez-Gerpe and Rey-Méndez, 1998; Bakovic et al., 2013; Pertsov et al., 2015), and increased inflammatory proteins and Hsp72. Specifically, IS increased blood glucose and plasma concentrations of corticosterone, CINC-1, IL-1β, IL-6, and IL-10. IS also increased MLN CINC-1, AT IL-1β; splenic CINC-1, Hsp72, and IL-1β; and liver CINC-1 and Hsp72. The anti-inflammatory effects of oral CBD-rich were tissue specific. Oral ingestion of CBD-rich hemp extract constrained stress-evoked increases in CINC-1, IL-6, and Hsp72 in MLNs, and CINC-1 in AT. In contrast, CBD-rich hemp extract had no effect on IS-induced increases in plasma corticosterone, CINC-1, IL-1β, IL-6, IL10, or blood glucose.

The hemp extract tested in this study was high in CBD (46.8 mg/g) and low in THC (2.0 mg/g). There is evidence that CBD impacts inflammation by modulating a wide array of systems and receptors. Based on studies testing purified CBD in inflammatory disease models, for example, there is evidence for the indirect and direct involvement of the endocannabinoid system, dopamine, transient receptor potential vanilloid type 1, peroxisome proliferator-activated receptors, and 5-HT_1A_ (Peres et al., 2018; Atalay et al., 2019; Jesus et al., 2019; Almeida and Devi, 2020; Sunda and Arowolo, 2020). In addition to CBD, the CBD-rich hemp also contained detectable levels of a variety of terpenes, including β-caryophyllene (193 ppm) and Myrcene (59 ppm). There is emerging *in vitro* and *in vivo* evidence that terpenes have biological effects (Rogerio et al., 2009; Liu et al., 2011; Jiang et al., 2015; Nuutinen, 2018; Hassanin et al., 2020; McDougall and McKenna, 2022). β-caryophyllene, for example, has anti-inflammatory and immunomodulatory effects (Gertsch, 2008); whereas myrcene has antioxidative cellular protective properties (Ciftci et al., 2011; Ciftci et al., 2014; Burcu et al., 2016). Using an *in vitro* model of glioblastoma, β-caryophyllene was reported to significantly down-regulate the NF-ĸB pathway and reduce the release of tumor necrosis factor-alpha (Irrera et al., 2020); and using an *in vivo* cerebral ischemia model, myrcene increased antioxidant defense systems, including glutathione, catalase, glutathione peroxidase and superoxide dismutase and decreased neuronal damage (Ciftci et al., 2011; Ciftci et al., 2014; Burcu et al., 2016).

Based on the literature, the CBD-rich hemp extract tested in this study was expected to have potent anti-inflammatory effects, however, most of the previous *in vivo* studies demonstrated the anti-inflammatory impacts of CBD and terpenes in inflammatory disease models or after injection of bacterial and viral pathogen-associated molecular patterns (PAMPs), including lipopolysaccharide or synthetic analogs of viral double-stranded RNA e.g. polyinosinic-polycytidylic acid (Atalay et al., 2019; Muthumalage and Rahman, 2019; Fitzpatrick et al., 2020; Trivedi et al., 2022). The current study, in contrast, explored the impacts of oral CBD-rich hemp extract on IS-induced inflammation. The sterile inflammatory response is triggered by stress-associated and not pathogen-associated signals including danger-associated molecular patterns (DAMPs), alarmins, and catecholamines (Johnson et al., 2005; Campisi et al., 2012; Cox et al., 2014). It is possible, therefore, that oral CBD-rich hemp extract more effectively impacts inflammatory pathways in models of disease rather than the exaggerated inflammatory response that is seen immediately following traumatic stress. CBD-rich hemp extracts may, therefore, be more useful at treating chronic low-grade inflammation that is often associated with stress and anxiety disorders following traumatic stressor exposure. While the anti-inflammatory effects found in this study were limited, they offered insight into the sterile inflammatory response in specific immune-related tissues where CBD may have the most potent effect after oral consumption.

Interestingly, there were sex differences in IS-evoked stress and inflammatory responses, as well as sex differences in the impacts of oral CBD-rich hemp extract. Consistent with previous reports, for example, IS stimulated a higher plasma corticosterone response in females compared to males (Nickerson et al., 2006). Less is known about sex differences in IS-evoked sterile inflammation. Although we found main effects of sex and IS for several tissue inflammatory proteins, only the mesenteric lymph nodes had a reliable sex by IS interaction. Specifically, males but not females had an IS-evoked increase in IL-6 in the MLNs. An interaction effect between sex, CBD, and stress for CINC-1 in AT revealed that oral CBD-rich hemp extract had differential impacts in females and males. Female rats that ingested CBD-rich hemp extract and were exposed to IS had increased levels of AT CINC-1 as compared to female rats exposed to IS in the Veh group. Male rats that consumed CBD-rich hemp extract from the HC group had higher levels of CINC-1 in AT as compared to males in the Veh HC group. Secondary analysis of the matched CBD-rich hemp cereal consumption groups confirmed the effect seen in the female groups; however, the oral CBD-rich hemp extract effect was now absent in the male rats. Additionally, both primary and secondary analyses revealed a stress-induced increase in AT CINC-1 only in female rats that ingested CBD-rich hemp extract. Taken together, these results show differences between female and male rats in response to stress and in home cage animals respectively. These differences could be due to sex differences in AT distribution, however, adipose distribution and how this affects CBD accumulation after oral administration in male versus female rats has not been documented.

Overall, the anti-inflammatory and stress-modulatory impacts of CBD-rich hemp extract were sexually dimorphic and tissue specific (i.e., AT and MLNs). The repeated oral consumption of CBD-rich hemp is unlikely to accumulate in plasma; however, it is feasible that CBD, terpenes, and their metabolites accumulate in lymphatic and fattier tissues. This is due to CBD’s lipophilic nature and that the MLNs drain the intestine (Zgair et al., 2016; Hlozek et al., 2017; Zgair et al., 2017; Child and Tallon, 2022). In fact, there is recent evidence that oral consumption of CBD accumulates in the MLNs and AT above immunomodulatory thresholds (Leighty, 1973; Devinsky et al., 2014; Chayasirisobhon, 2020; Jewell et al., 2022). Many variables contribute to the complex pharmacodynamics of orally ingested CBD, including sex differences in pharmacokinetics. In the current study, we reported that oral ingestion of CBD-rich hemp extract increased COOH-CBD in the plasma more in females than in males. This finding could reflect differences in metabolism and clearance. In addition, Child and Tallon, 2022 recently reported that when compared to male rats, female rats had higher CBD concentrations in liver and muscle tissue after ingesting oral CBD (30.0 mg/kg) and this effect was dose-dependent (Child and Tallon, 2022). Interestingly, even though male rats in the CBD-rich hemp extract group consumed fewer cereal pieces than female rats, the anti-inflammatory effects seen in the MLNs and AT of male rats remained after secondary analysis of the matched CBD-rich hemp cereal consumption groups, suggesting that even small amounts of CBD can exert notable effects in some tissue. The complexity of the results from this study highlights the need for additional, controlled, preclinical studies that test the stress-modulatory and anti-inflammatory impacts of oral CBD-rich hemp extract.

## 5 Limitations

A weakness of this study was the variability of the voluntary dosing between females and males, potentially confounding our results due to differences in the ingestion of CBD-rich hemp extract. A secondary analysis was performed to address this issue. Additionally, this study had no measures of concentrations of CBD, terpenes, and their metabolites in tissues.

## 6 Conclusion and future directions

There are several strengths of the current study that add to the significance and relevance of our results. First, we tested stress-evoked, not pathogen-associated, inflammatory responses. Stress-induced sterile inflammatory processes are likely contributing to inappropriate inflammation commonly associated with anxiety, insomnia, and pain. These conditions are among the top reasons for CBD treatment, each of which is exacerbated by stress and associated with inappropriate inflammation (Salim et al., 2012; Fleshner, 2013; Corroon and Phillips, 2018; Lurie, 2018; Devine et al., 2019; Wheeler et al., 2020; Moltke and Hindocha, 2021; Cavalcante et al., 2022; Uy et al., 2022). Second, we measured pro-inflammatory cytokines (IL-1β, IL-6), chemokines (CINC-1), anti-inflammatory cytokines (IL-10), and cellular stress proteins (Hsp72) in plasma and tissues. This is important because it is feasible, based on our results, that differences in oral CBD-rich hemp pharmacokinetics contribute to tissue specific effects. And third, we tested a complex formulation of CBD-rich hemp that contained terpenes thought to enhance therapeutic benefits; used voluntary daily oral ingestion; and tested females and males. Our study design, therefore, better reflects the way people use CBD to self-manage stress and anxiety. Possible future directions are to investigate the accumulation of CBD and its metabolites in stress and immune-responsive tissues in both female and male rats over the course of oral ingestion. Finally, a non-stressful oral dosing protocol that ensures equal dosing among groups should be implemented in future studies to better examine any sex specific effects of CBD-rich hemp extract on stress induced sterile inflammation.

## Data Availability

The raw data supporting the conclusion of this article will be made available by the authors, without undue reservation.
