# Supplementary material for: Oral CBD-rich hemp extract modulates sterile inflammation in female and male rats
Source: Front Physiol. 2023 May 18;14:1112906. doi: 10.3389/fphys.2023.1112906 (PMC10234154; doi:10.3389/fphys.2023.1112906)
Supplement: Supplementary file 1 [file Image1.pdf]

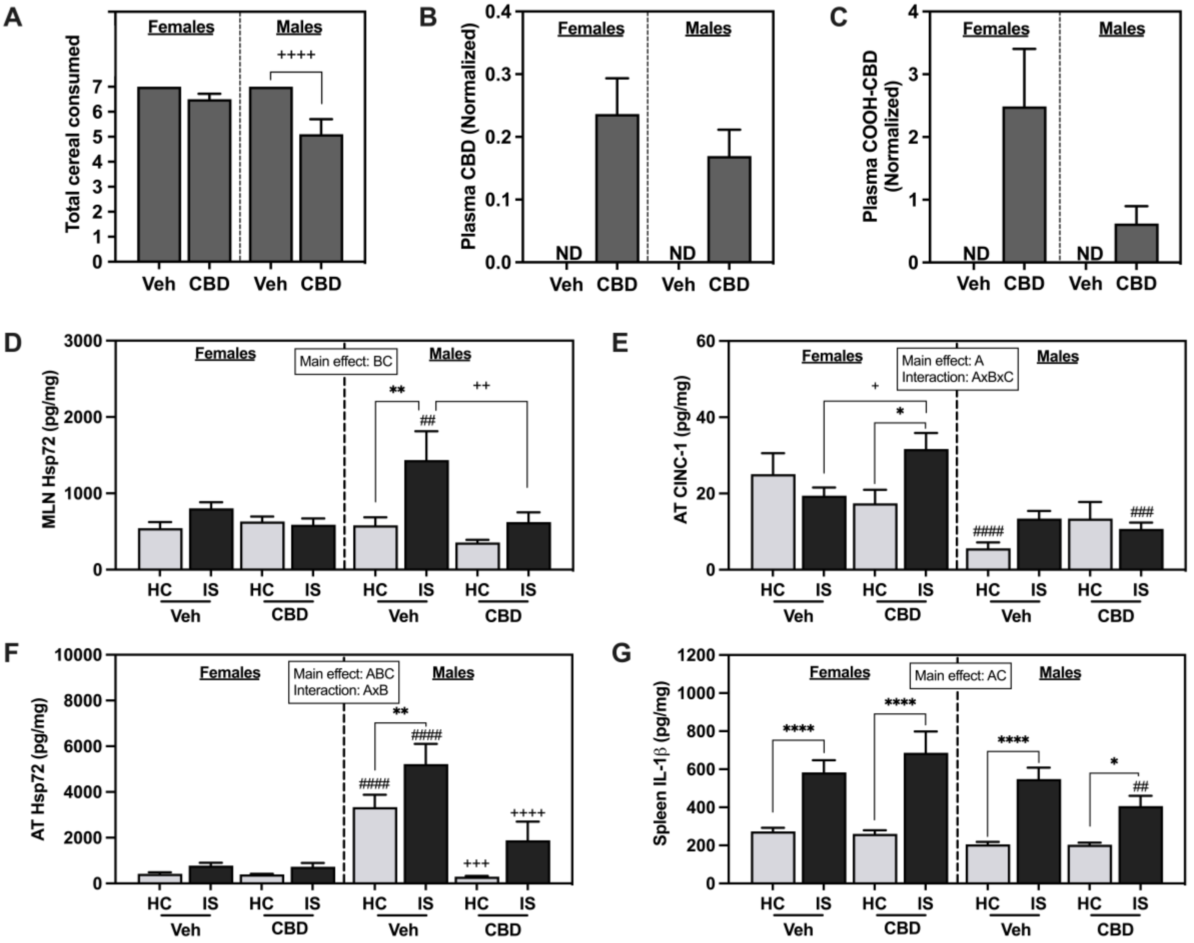

Supplementary Figure 1. Total voluntary consumption of coconut oil vehicle (Veh) or CBD-rich hemp extract (CBD) treated cereal **(A)**. All female and male rats assigned to the vehicle group consumed every piece of coconut oil-treated cereal they were offered. Concentrations of normalized plasma **(B)** cannabidiol (CBD), and **(C)** COOH-CBD (unpaired T-test effect, female vs male); ND = not detectable. Concentrations of **(D)** Mesenteric lymph node (MLN) Heat shock protein 72 (Hsp72), **(E)** Adipose tissue (AT) CINC-1, **(F)** AT Hsp72, and **(G)** spleen IL-1β. Females are graphed to the left of the dashed line; males are graphed to the right of the dashed line. Data are presented as mean ± SEM; Symbols, Figure A: $^{++++}p < 0.0001$ (Dunn's *post hoc* effect of CBD); Figures D-G: $^{A}p < 0.05$ (main effect of sex); $^{B}p < 0.05$ (main effect of CBD); $^{C}p < 0.05$ (main effect of IS); $^{\#\#}p < 0.01$, $^{\#\#\#\#}p < 0.0001$ (Fisher's LSD *post hoc* effect of Sex); $^{+}p < 0.05$, $^{++}p < 0.01$, $^{+++}p < 0.001$, $^{++++}p < 0.0001$ (Fisher's LSD *post hoc* effect of CBD); $^{*}p < 0.05$, $^{**}p < 0.01$, $^{****}p < 0.0001$ (Fisher's LSD *post hoc* effect of IS). Vehicle group = Veh,

Cannabidiol-rich hemp extract group = CBD, unstressed home cage controls = HC, inescapable tail shock = IS.
